# Gum tragacanth, a novel edible coating, maintains biochemical quality, antioxidant capacity, and storage life in bell pepper fruits

**DOI:** 10.1002/fsn3.4052

**Published:** 2024-02-27

**Authors:** Mohammad Reza Zare‐Bavani, Mostafa Rahmati‐Joneidabad, Hossein Jooyandeh

**Affiliations:** ^1^ Department of Horticultural Science, Faculty of Agriculture Agricultural Sciences and Natural Resources University of Khuzestan Mollasani Iran; ^2^ Department of Food Science, Faculty of Animal and Food Science Agricultural Sciences and Natural Resources University of Khuzestan Mollasani Iran

**Keywords:** antioxidant enzymes, lipid peroxidation, marketability, total carotenoid, total phenol

## Abstract

Bell pepper fruits (*Capsicum annuum* L.) are prone to both physiological and pathological deterioration following harvest, primarily due to their high metabolic activity and water content. The storage of bell peppers presents several challenges, including weight loss, softening, alterations in fruit metabolites and color, increased decay, and a decline in marketability. The application of edible coatings (ECs) is one of the environmentally friendly technologies that improves many post‐harvest quantitative and qualitative characteristics of products. This research investigated the impact of different levels of gum tragacanth (GT) coating (0, 0.25, 0.5, 1, and 2%) on the physiological and biochemical traits of stored bell pepper fruits (BPFs) (8 ± 1°C, 90–95% RH) for 28 days. The results showed the positive effect of coating treatments with higher concentrations of GT, up to 1%. Increasing the concentration of GT to 2% decreased the marketability and quality characteristics of fruits compared to 1% GT. After storage, the physiological weight loss of the fruits treated with 1% GT (10.46%) was lower than that of the uncoated fruits (18.92%). Furthermore, the coated fruits (1% GT) had more firmness, total phenol content, ascorbic acid, and titratable acidity content than uncoated fruits during storage. At the end of storage, the coated BPFs with 1% GT showed higher SOD (97.02 U g^−1^), CAT (24.38 U g^−1^) and POD (0.11 U g^−1^) activities and antioxidant capacity (81.74%) as compared to other treatments. Total soluble solids, total carbohydrates, total carotenoids, pH, malondialdehyde, and electrolyte leakage content increased in coated fruit during storage but were significantly lower than in uncoated fruits. Moreover, the samples coated with GT (1%) maintained good marketability (about 75%), while the marketability of the control (about 40%) was unacceptable. The study shows that GT (1%) coating can be a promising novel treatment option for increasing the storage quality of BPFs.

## Graphical abstract

This study investigated the impact of varying levels of gum tragacanth (GT) coating on the quality of stored sweet pepper fruits over 28 days. Results indicated that GT concentrations up to 1% positively influenced fruit quality, while 2% concentrations had negative effects on marketability and overall quality. Fruits coated with 1% GT exhibited lower weight loss, more firmness, and higher levels of phenols, ascorbic acid, titratable acidity, SOD, CAT, POD activities, and antioxidant capacity compared to other treatments.

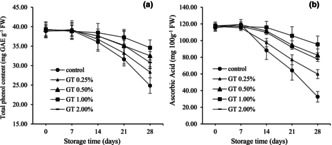

## 1 INTRODUCTION

Bell peppers (*Capsicum annuum* L.) are one of the most significant commercial crops, cultivated and consumed on an enormous scale. BPFs are one of the most widely consumed foods worldwide due to their attractive colors, strong taste, high nutrients, bioactive compounds such as vitamins C, A, B, and E, carotenoids, phenolic compounds, as well as antioxidant and antimicrobial substances (Kumar et al., 2021; Rodríguez et al., 2020). It has been reported that the global cultivation of bell peppers spans an area of 1.99 million hectares, with a total production of 38 million metric tons. This crop is grown in 126 countries across the world (Tiamiyu et al., 2023). More than 70% of greenhouse products are dedicated to vegetables. Among them, greenhouse bell peppers, with 270,000 tons of annual production, are the third most important greenhouse product in Iran (MAJ, 2023) and many other countries, which has found a good export market. Despite the high quality and compliance with health conditions in greenhouse pepper production, pepper fruits usually suffer from many post‐harvest problems, such as quality loss and reduced shelf life, chilling injury at temperatures below 7°C, susceptibility to diseases, and shriveling, along with rapid weight loss (Edirisinghe et al., 2014; Kumar et al., 2021; Martinez‐Romero et al., 2006; Xing et al., 2011).

Packaging plays a crucial role in the food industry, ensuring the protection and preservation of products. Edible coatings, as a specific type of packaging, offer unique advantages in terms of extending shelf life, enhancing quality, and reducing waste (Han, 2014). One of the successful methods used in the storage and handling of fruit and vegetable products to preserve the post‐harvest quality and quantity of these products is the use of edible coatings (ECs) (Andriani & Handayani, 2023; Dhall, 2013; Salehi, 2020). By creating a semi‐permeable membrane layer, ECs reduce gas exchange with the environment, moisture loss, respiration, physiological disorders (such as browning, color change, taste change, and loss of nutrients), and microbial activity, and thus increase the shelf life and better preserve the quantitative and qualitative traits of these products (Ali et al., 2015; Andriani & Handayani, 2023; Nasiri et al., 2017; Salehi, 2020). Another advantage of ECs is their naturalness. They can be consumed with fruits and vegetables (Andriani & Handayani, 2023). Various compounds are used as ECs and are usually protein‐, lipid‐, or polysaccharide‐based compounds (Andriani & Handayani, 2023; Dhall, 2013; Salehi, 2020). Some ECs used to maintain the freshness and quality of fruits and vegetables are derived from hydrocolloids, including gums, chitosan, and alginate (Valero et al., 2013; Raghav et al., 2016). Among ECs, polysaccharide‐based ones have the most application to increase the shelf life of fruits and vegetables (Nasiri et al., 2017).

Gum tragacanth (GT) is one of the three important and abundant secretory gums secreted spontaneously or by scratching on different species of *Astraglus* (Nasiri et al., 2017). This natural polysaccharide is safe and non‐toxic for food consumption. It is also stable and environmentally friendly in a wide pH range, and is mainly found in the mountainous and semi‐desert regions of Iran and Asian countries (Hemmati & Ghaemy, 2016). GT has been recognized as an approved additive by the European and American Scientific Committee on Food for several decades (Ghayempour et al., 2015), and it is widely utilized worldwide as a thickener, stabilizer, emulsifier, fat substitute, and binding agent in food and pharmaceutical systems (Kurt et al., 2016). GT contains natural antimicrobial and antioxidant compounds, and its use as an EC increases the shelf life and preserves the quality of edible mushrooms (Nasiri et al., 2017, 2019). It has been reported that GT maintains the post‐harvest quality of apricots by reducing oxidative stress (Ali et al., 2015). Researchers have shown that GT increases the shelf life of apricots (Ziaolhagh & Kanani, 2021) and preserves the storage quality of tomatoes (Jahanshahi et al., 2023).

Currently, the advantages of chitosan as an edible coating for bell peppers have been scientifically substantiated, establishing it as one of the most effective options (Gholamipour Fard et al., 2009; Kumar et al., 2021; Taheri et al., 2020; Xing et al., 2011). Nonetheless, the issue of affordability remains a challenge, particularly in developing and third‐world nations. GT possesses desirable physical and chemical properties for use as a coating. It is widely available and more cost‐effective compared to other coatings, such as chitosan. The objective of this study was to examine the impact of GT, used without any additives, as a tasteless and colorless food coating, on the shelf life, as well as the physiological and biochemical characteristics, of greenhouse BPFs.

## 2 MATERIALS AND METHODS

### 2.1 Sampling

The study utilized sweet red bell peppers (*Capsicum annuum* L.) that were cultivated in a greenhouse (32°19′83″N, 48°72′19″E). These BPFs were selected based on their uniform size and absence of contamination, and they were harvested at the breaking point stage. After the initial cooling stage (8°C, 12 h) (Xing et al., 2011), the BPFs were transported to the laboratory (the Horticultural Science Department of Agricultural Sciences and Natural Resources, University of Khuzestan, Ahvaz, Iran).

### 2.2 Preparation of raw materials and experimental design

All the chemicals utilized in the research were obtained from Sigma Chemical Corporation (St. Louis, MO). GT solutions (0.00, 0.25, 0.50, 1.00, and 2.00% W:V) were prepared following the methodology outlined by Ziaolhagh and Kanani (2021). To elaborate, 2.5, 5.0, 10.0, and 20.0 g of GT powder were dissolved in 1000 mL of distilled water at 40°C for 10 min. The control group was prepared using distilled water. Glycerol (1%, as a softener) and Tween 80 (0.05%, as an emulsifier) were added to the solutions, and the mixture was stirred for 30 min with an electric stirrer to achieve a uniform solution. The solutions were then refrigerated for 24 h before being utilized for the treatments.

Prior to treatment, the fruits were disinfected with a 0.05% sodium hypochlorite (NaClO) solution for 3 min and subsequently air‐dried at room temperature. The final coating solutions were homogenized with a stirrer for 30 min prior to application, and the BPFs were immersed in varying amounts of GT (0.00, 0.25, 0.50, 1.00, and 2.00%) for 5 min. Subsequently, the coated BPFs were dried, packed in polystyrene boxes, and stored at 8 ± 1°C with 90–95% relative humidity (Ullah et al., 2017) for a duration of 28 days. At seven‐day intervals, the BPFs were removed from cold storage to assess physiological weight loss, fruit firmness, total soluble solids, titratable acidity, total carbohydrates, total phenol, ascorbic acid, total carotenoid, marketability, total antioxidant capacity, antioxidant enzyme activity, lipid peroxidation, and membrane leakage.

### 2.3 Physicochemical measurements

#### 2.3.1 Physiological weight loss

Physiological weight loss was measured according to the method described by Samira et al. (2013). The stored BPFs were weighed at the beginning and at seven‐day intervals for 4 weeks. Total weight loss was determined as the difference between the initial weight and each sampling time, and was calculated as follows:

$$\text{Physiological Weight Loss}\;\left(\%\right)=\left(\frac{W_{i}-W_{f}}{W_{i}}\right)\times 100$$

where *W*_*i*_ = the initial weight of the sample, and *W*_*f*_ = the final weight of the sample at each measurement time.

### 2.4 Fruit firmness

The texture firmness of BPFs was measured using a texture analyzer (Stable Micro System Texture Analyzer, TA, XT2i, UK). The fruits were analyzed with a 2 mm diameter probe at a speed of 10 mm min^−1^ to pierce the equatorial location of the fruit (five points), and the results were expressed as the maximum penetration force (N) during tissue breakage (Kumar et al., 2021).

### 2.5 The percentage of marketable fruits

The visual assessment of the marketable fruit percentage was conducted based on the outward quality of the fruits. Initially, the samples were placed in sealed plastic containers with random numbers assigned to them. These containers were then distributed to a group of trained individuals known as the test panel, consisting of 20 panelists. The quality of the fruits was evaluated using a ranking system ranging from 1 to 9, which determined their suitability for the market. Parameters such as size, color, tissue firmness, and the presence of fungal or bacterial decay were considered during the visual assessment. Fruits with a score of five or above were considered marketable, while those with a score below five were deemed unmarketable (Samira et al., 2013).

### 2.6 Total soluble solids (TSS)

Three pieces of the BPFs (100 g) from each replication and three replications per treatment were randomly taken and mixed into homogeneous solutions using an electric mixer. This extract was used for TSS, TA, and pH measurements. The total dissolved solids of pepper extracts were measured using a portable refractometer (Milwaukee model 871, Romania). The device was calibrated using distilled water. The results were expressed in °Brix (Kumar et al., 2021).

### 2.7 Titratable acidity (TA)

The prepared extract (10 mL) was passed through a funnel containing Whatman No. 1 filter paper for filtration. An equal volume of distilled water was combined with the filtered extract. The resulting solution was immediately titrated using NaOH (0.1 N) until reaching a pH of 8.2. The amount of NaOH used in the following formula was used to calculate TA and was reported as a percentage in terms of citric acid (Kumar et al., 2021):

$$\text{Titratable acidity}\left(\%\right)=\left(\frac{V_{\text{NaoH}}\times 0.064}{10}\right)\times 100$$

where *V*_NaoH_ is the volume of sodium hydroxide used, and 0.64 is the conversion factor for citric acid.

### 2.8 Maturity index (TSS/TA)

The maturity index was calculated by dividing the TSS value of each sample by the percentage of TA.

### 2.9 pH

To begin, 20 mL of the prepared extract was carefully transferred into a beaker. Subsequently, 100 mL of distilled water with a pH of 7 was added to the beaker. Throughout the measurement process, an electric stirrer was utilized to stir the mixture. The pH of the extracts was then determined using a digital pH meter (InoLab 7110, Germany). Each measurement consisted of two samples per replicate, ensuring a more comprehensive assessment of the pH values.

### 2.10 Total soluble carbohydrates (TSC)

Total carbohydrate content was determined following the method outlined by Dubois et al. (1956). 100 mg of coated and uncoated BPFs were mixed with 5 mL of ethanol (80%) at 80°C. The mixture was then subjected to centrifugation at 7155 *g* for 10 min. By repeating these steps, the final volume was increased to 10 mL using 80% ethanol. Two milliliter of the resulting extract were transferred to a test tube, to which 1 mL of a 5% phenol solution was added. Immediately after, 5 mL of concentrated sulfuric acid was introduced, and the mixture was vigorously vortexed for 1 min. Subsequently, the test tube was left at room temperature for 10–15 min, resulting in the development of a brick‐brown color. Standard glucose was employed for calibration purposes. The absorbance of the samples was measured at 490 nm, and the total carbohydrate content was calculated in grams per gram of dry weight of the sample (Dubois et al., 1956).

### 2.11 Total carotenoid content (TCC)

The determination of total carotenoid content (TCC) followed the method described by Burgos et al. (2009). Two grams of treated and untreated fruit tissue were homogenized with acetone. The homogenization process was repeated until the samples became colorless. Petroleum ether was added to the extracts, and then they were washed with water to remove the remaining acetone. Butylated hydroxytoluene was added to prevent the degradation of carotenoids. Saponification was done with methanolic potassium hydroxide (10%) in a volume equal to the extract in a dark environment at room temperature. The absorption values of the samples were measured at 450 nm, and TCC was determined by applying the appropriate extinction coefficient for the carotenoid mixture, which was set at 2500.

### 2.12 Total phenol content (TPC)

The total phenol content (TPC) was determined following the methodology described by Ghasemnezhad et al. (2011). A gram of powdered plant sample was extracted with 10 mL of cold methanol solvent using liquid nitrogen. Subsequently, 125 μL of the methanolic extract was mixed with 375 μL of distilled water and 2.5 mL of a 10% Folin–Ciocalteu reagent in a test tube. The mixture was then kept in the dark at room temperature for 6 min. Next, 2 mL of a 7.5% sodium carbonate solution was added to neutralize the reaction. The samples were left in the dark for 90 min at room temperature, and the resulting solution's absorbance was measured at 765 nm using a spectrophotometer. A standard curve was constructed using gallic acid (ranging from 50 to 1000 mg/L). The TPC was expressed as milligrams of gallic acid per 100 g of fresh tissue.

### 2.13 Ascorbic acid content (AsA)

The ascorbic acid content (AsA) of fruit samples was measured using the method of Klein and Perry (1982), with minor modifications. Five grams of the pepper samples were extracted with 50 mL metaphosphoric acid (1% W:V). Then, the resulting extract was centrifuged by a refrigerated centrifuge at 5000 *g* for 5 min. Next, 1 mL of the supernatant was mixed with 9 mL of dichlorophenol indophenol (DCIPP) solution (0.05 mM), and immediately, the absorption of the samples was read using a spectrophotometer at 515 nm. L‐ascorbic acid was used to prepare the standard. AsA was calculated in milligrams per 100 grams of fresh weight (Klein & Perry, 1982).

### 2.14 Activity of antioxidant enzymes (superoxide dismutase (SOD), peroxidase (POD), and catalase (CAT))

The extraction and activity assessment of SOD, CAT, and POD enzymes were conducted following the methodology outlined by Xing et al. (2011).

### 2.15 Extraction

Fruit pulp samples (2 g) were homogenized with 10 mL of sodium phosphate buffer solution (25 mM, pH = 7.8) containing PVPP (0.8 g L^−1^) and EDTA (1 mM). The mixture was then subjected to centrifugation at 12,000 *g* for 20 minu at 4°C. The successive supernatant was used as an extract to measure antioxidant enzymes.

To determine SOD enzyme activity, 0.1 mL of the enzyme extract was mixed with 2.9 mL of sodium phosphate buffer (50 mM, pH = 7.8) containing methionine (13 mM), nitroblue tetrazolium (75 μM NBT), riboflavin (2 μM), and EDTA (10 μM). The resulting mixture was treated for 10 min with a light intensity of 60 mol m^−2^ s^−1^, and the absorbance was recorded at 560 nm. The blank solution was the reaction mixture that was kept in the dark. One enzyme unit was considered equivalent to a volume of an enzyme that causes 50% inhibition of NBT reduction at 560 nm. Finally, the SOD activity was reported as U g^−1^ FW.

One milliliter of the enzyme extract was mixed with 1 mL of sodium phosphate buffer (50 mM, pH = 7.0) and 1 mL of H_2_O_2_ (40 μM) to determine the CAT activity. The decrease in absorbance was measured at 240 nm. The amount of CAT activity was calculated according to the following formula and finally reported as U g^−1^ FW.

$$U=0.1\times \Delta A_{240\mathrm{nm}}\;\mathrm{per}\;\min$$

A volume of 0.5 mL of the enzyme extract was combined with 2 mL of sodium phosphate buffer (100 mM, pH = 6.4) containing guaiacol (8 mM) to assess the activity of the POD enzyme. The mixture was then incubated at a temperature of 30°C for a duration of 5 min. Following this, 1 mL of H_2_O_2_ (24 mM) was added, and the increase in absorbance at a wavelength of 460 nm was measured at 30‐second intervals for a total of 2 min. The POD enzyme activity was calculated using the provided formula and reported as U g^−1^ FW.

$$U=0.01\times \Delta A_{470\mathrm{nm}}\;\mathrm{per}\;\min$$

### 2.16 Total antioxidant capacity (TAC)

The TAC of fruits was measured by inhibiting soluble free radicals 2, 2 diphenyl‐1‐picrylhydrazyl (DPPH) (Ghasemnezhad et al., 2011). For this purpose, using a sampler, 50 μL of pepper extract were poured into small Falcon tubes, and 950 microliters of DPPH solution (6.25 × 10^−5^ M) were added to it and vortexed. The resulting solution was stored at room temperature in the dark. After 15 min, the absorbance of the samples was read with a spectrophotometer (model PG Instrument +80, Leicester, UK) at 515 nm. The TAC was expressed based on the reduction of absorbance compared to the control in terms of the percentage of DPPH inhibitory power using the following formula:

$$\text{DPPHsc}\left(\%\right)=\left(\frac{A_{\text{cont}}-A_{\text{samp}}}{A_{\text{cont}}}\right)\times 100$$

where %DPPHsc = inhibition percentage, *A*cont = DPPH absorption rate, and Asamp = absorption rate (sample + DPPH).

### 2.17 Lipid peroxidation

The extraction and determination of membrane lipid peroxidation were done as described by Xing et al. (2011). The lipid peroxidation was measured and expressed based on the amount of malondialdehyde (MDA) produced. The fleshy tissue of pepper fruits (4 g) was homogenized with 20 mL of trichloroacetic acid (10%). The resulting mixture was then subjected to centrifugation at 5000 *g* for 10 min. One milliliter of the supernatant was combined with 3 mL of 0.5% thiobarbituric acid dissolved in 10% trichloroacetic acid. Next, the reaction mixture was incubated at 95°C for 20 min, rapidly cooled, and then centrifuged at 10,000 *g* for 10 min to obtain the sediment. The absorbance of the samples at 532 nm and 600 nm was recorded. The amount of MDA was computed using the extinction coefficient $155\;{\mathrm{mM}}^{-1}{\mathrm{cm}}^{-1}$ and the provided formula:

$$\mathrm{MDA}\;\left(\text{μmol}\;g^{-1}\;\mathrm{FW}\right)=\left({\mathrm{OD}}_{532}–{\mathrm{OD}}_{600}\right)\times V_{t}\times V_{r}\times 1000/\left(V_{m}\times m\times 155\right)$$

In this context, *V*_t_ represents the total volume of the extract solution, *V*_r_ signifies the total volume of the reaction mixture, *V*_m_ corresponds to the volume of the extract solution within the reaction mixture, and *m* denotes the mass of the sample.

### 2.18 Relative membrane permeability (RMP)

The measurement of membrane permeability, also known as the relative leakage rate, was carried out following the procedure outlined by Xing et al. (2011). Fifty‐mm thick discs were prepared from the middle part of the pepper fruits. These discs were thoroughly washed three times with deionized water to remove any electrolyte residue on the surface. Afterward, the discs were gently dried using filter paper. For each measurement, ten of these discs were placed in glass vials with lids, each containing 30 mL of deionized water. The vials were then placed on a rotary shaker at a temperature of 25°C for a duration of 30 min. The electrical conductivity of the solution inside the vials ($L_{t}$) was determined using an EC meter (model DDSJ‐308A, Shanghai Precision & Scientific Instrument Co., Ltd., Shanghai, China). Subsequently, the vials containing the samples and solution were boiled for 10 min, rapidly cooled, and the final electrical conductivity ($L_{0}$) was measured. The relative leakage rate was calculated as the percentage of total electrolytes using the following formula:

$$\mathrm{RMP}\left(\%\right)=\left(L_{t}/L_{0}\right)\times 100$$

### 2.19 Data analysis

The experiment was conducted using a completely randomized design in the form of a split plot design over time with 3 replications. Each replication consisted of 10 fruits. The first experimental factor considered was the 5 levels of edible coating (0.00, 0.25, 0.50, 1.00, and 2.00), while the second experimental factor was the sampling time (0, 7, 14, 21, and 28 days). Fruit weight loss was measured at different time points using 10 fruits separately, with three replicates. Statistical analysis of the data, including tests for normality of data distribution, analysis of variance, and mean comparisons, was performed using SAS software (Version 9.1). Graphs were created using Excel software (2013). The comparison of means was conducted using the LSD test at the 5% significance level, and mean values were reported as mean ± standard deviation (SD) based on three samples (*n* = 3).

## 3 RESULTS

### 3.1 Physiological weight loss (PWL)

**FIGURE 1:**
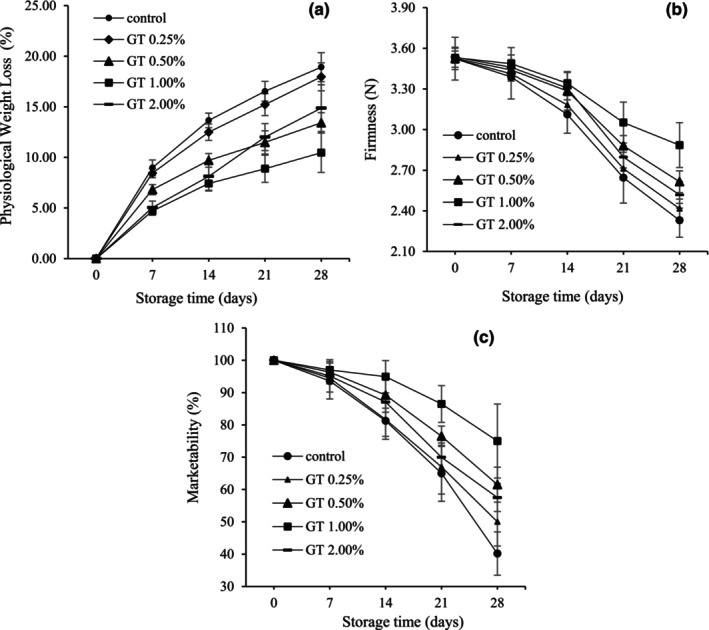
Cumulative weight loss (%) (a), fruit firmness (b), and marketability (%) (c) in pepper fruits coated with GT (0.00, 0.25, 0.50, 1.00, and 2%). The fruits were stored at 7°C, 95% RH, for 28 days. LSD_0.05_ indicates the least significant difference (*p* < .05). Vertical bars represent the standard deviation (SD) (*N* = 3).

In this study, we examine the impact of GT coatings as natural protectors on the cumulative PWL in BPFs. Weight changes during storage show the effectiveness of coating methods compared to uncoated peppers (Figure 1a). After 28 days, the peppers with GT coating (1%) and without coating showed the lowest (10.46%) and highest (18.92%) PWL, respectively. Increasing the concentration of GT from 0.25 to 1% slowed down the PWL process (Figure 1a). However, increasing the GT concentration to 2% during the first and second weeks of storage did not show significant differences compared to the 1%‐coated fruits. Nonetheless, in the third and fourth weeks of storage, it led to an increase in PWL. Furthermore, among the samples of peppers with gum coatings, the sample with 0.5% GT showed more PWL (Figure 1a).

### 3.2 Fruit firmness

The results showed a substantial interaction (*p* ≥ .01) between coating treatment and storage duration on fruit firmness. Figure 1b shows that throughout the storage period, the fruit firmness decreased in all treatments. Decreasing firmness was more evident for uncoated and GT‐coated (0.25%) fruits. Increasing the coating concentration up to 1% improved the fruit firmness in storage duration so that the treatments of 1% GT had the highest (2.89 N) and uncoated fruits had the lowest (2.33 N) firmness. Increasing the level of gum coating by 2% GT decreased the fruit firmness as compared to samples having 0.5 and 1% GT.

### 3.3 Marketability

**FIGURE 2:**
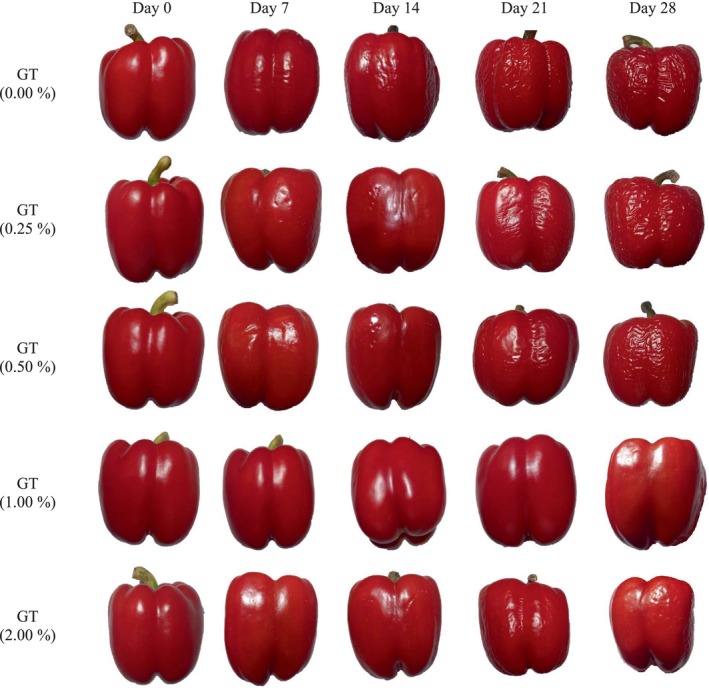
The effect of different concentrations of gum tragacanth as an edible coating on red bell pepper fruit (*Capsicum annuum* L.) at various storage times.

Figure 1c shows the marketability of bell pepper fruits with different coatings and without coating during 28 days of storage. Coated BPFs presented higher acceptability than uncoated peppers. The percentages of marketability of peppers treated with 1% GT and uncoated fruits at the end of storage were 75 and 40.17%, respectively. Similar to the decrease in fruit weight and firmness, increasing the concentration of gum coating by 2% decreased marketability compared to 1 and 0.5% treatments. As depicted in Figure 2, the decline in marketability was primarily manifested as wrinkles and roughness.

### 3.4 Total soluble solids (TSS)

**FIGURE 3:**
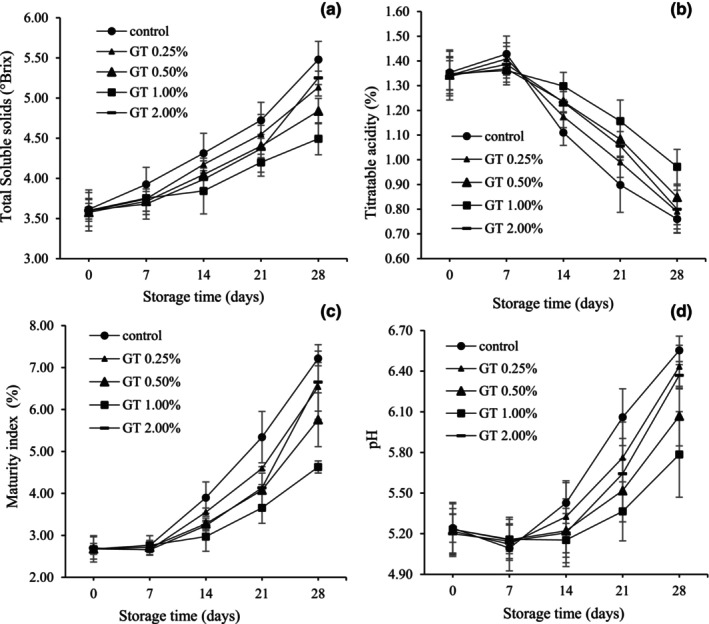
Total soluble solids (Brix°) (a), titratable acidity (%) (b), maturity index (c), and pH (d) in pepper fruits coated with GT (0.00, 0.25, 0.50, 1.00, and 2.00%). The fruits were stored at 7°C, 95% RH, for 28 days. LSD_0.05_ indicates the least significant difference (*p* < .05). Vertical bars represent the standard deviation (SD) (*N* = 3).

Figure 3a shows changes in TSS in BPFs during the storage period. The TSS of the control showed the highest increase with storage time, while the fruits coated with GT (1%) showed a relatively smaller increase. Increasing the concentration of gum coating up to 1% had more reducing effects on increasing TSS, but its rise to 2% showed the opposite impacts (Figure 2a).

### 3.5 Titratable acidity (TA)

As seen in Figure 3b, TA on the seventh day of storage in uncoated BPFs increased compared to the first day of measurement and showed a significant decrease until the 28th day of storage. No substantial differences were identified in the amount of TA in coated BPFs on the first and seventh days of storage, but after that, a decreasing trend occurred in all treatments, and the decreasing trend in the 1% GT was significantly lower than the other treatments. Storage of the uncoated fruits led to a faster reduction of pepper TA than coated storage. Increasing the coating concentration to 1% decreased the TA reduction process, but the 2% concentration again caused a further increase in TA (Figure 3b).

### 3.6 Maturity index (MI)

The values of TSS/TA, which express the maturity index (MI), or the degree of ripening, were not different on the seventh day compared to the first day, but these values boosted with increasing the storage time in all treatments. The amount of increase in MI in BPFs treated with GT was lower than that of uncoated fruits, and increasing the level of coatings up to 1% had a more inhibitory effect on this index, so fruits coated with 1% GT had the lowest MI values recorded at the measurement times (Figure 3c). Increasing the coating concentration to 2% increased the MI values more than the coating concentration of 0.5% (Figure 3c).

### 3.7 pH

The pH of uncoated samples decreased during storage until the seventh day and then increased until the end of storage (*p* < .05) (Figure 3d). No substantial changes were recognized in the pH of fruits coated with 0.25% GT compared to the control during storage. The lowest increase in pH at the end of storage was related to 1% TG‐coated fruits. Fruits coated with concentrations of 0.50, 1.00, and 2.00% of GT indicated no differences (*p* < .05) in terms of pH until the 14th day of storage compared to the 7th and 1st days (Figure 3d).

### 3.8 Total soluble carbohydrates (TSC)

**FIGURE 4:**
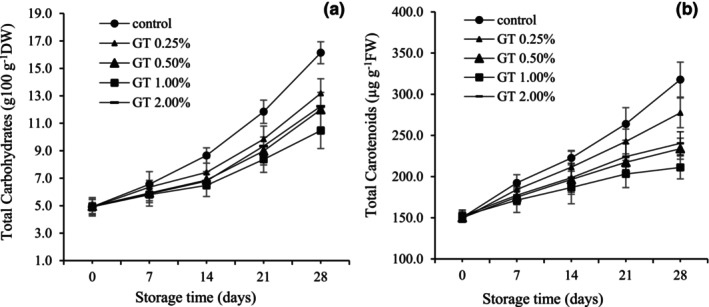
Total carotenoids (a) and total soluble carbohydrates (b) in pepper fruits coated with GT (0.00, 0.25, 0.50, 1.00, and 2.00%). The fruits were stored at 7°C, 95% RH, for 28 days. LSD_0.05_ indicates the least significant difference (*p* < .05). Vertical bars represent the standard deviation (SD) (*N* = 3).

The content of TSC in uncoated and coated fruits increased during storage (Figure 4a). However, this increase was less in coated fruits. The lowest increase was 2.13 times in 1% GT‐coated fruits, and the highest was 3.3 times in uncoated fruits. Increasing the coating concentration to 1% decreased the rise, but the coating concentration of 2% again increased the TSC of the fruit (Figure 4a).

### 3.9 Total content of carotenoids (TCC)

Figure 4b shows changes in the TCC of pepper fruits during storage time. Uncoated fruits had the highest TCC value during storage (Figure 4b). This increase was slower in the fruits coated with GT, and with increasing the coating concentration to 1%, this trend showed a further decrease. The lowest increase in TCC in 1% GT‐coated fruits was about 1.4 times, and the highest in uncoated fruits was about 2.9 times, almost twice that of fruits coated with 1% GT.

### 3.10 Total phenolic contents (TPC)

**FIGURE 5:**
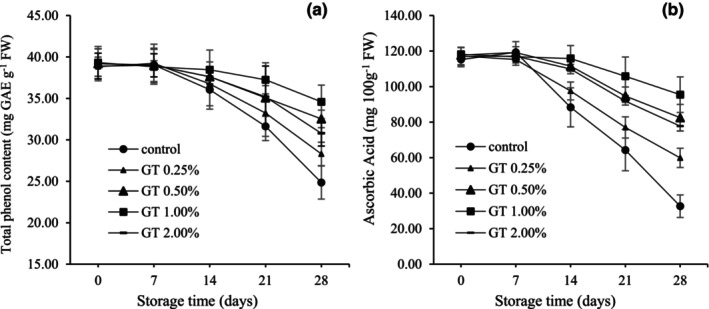
Total phenol (mg GAE g^−1^ FW) (a) and ascorbic acid (mg 100 g^−1^ FW) (b) in pepper fruits coated with GT (0.00, 0.25, 0.50, 1.00, and 2.00%). The fruits were stored at 7°C, 95% RH, for 28 days. LSD_0.05_ indicates the least significant difference (*p* < .05). Vertical bars represent the standard deviation (SD) (*N* = 3).

Figure 5a illustrates the changes in TPC of uncoated and coated BPFs during storage. The graph shows that TPC decreased with increasing storage time from day 14 in all treatments. This reduction was considerably higher in uncoated fruits, but the process was slower in coated fruits. Over time, this difference between the treatments increased. In 1% GT‐coated fruits, the reduction in TPC showed the lowest level among the treatments in all stages of storage, so there was no substantial difference from the beginning of storage until the 14th day. At the end of storage, TPC decreased in the control (24.85 mg GAE g^−1^ FW), the fruits coated with 0.25 (28.30 mg GAE g^−1^ FW), 2.00 (30.79 mg GAE g ^−1^ FW), 0.50 (32.53 mg GAE g^−1^ FW), and 1.00% GT (34.59 mg GAE g^−1^ FW), respectively (Figure 5a).

### 3.11 Amount of ascorbic acid (AsA)

As presented in Figure 5b, the highest amount of AsA in different BPF treatments was observed on the 7th day (117 mg 100^−1^g FW). However, there was no significant difference from the first day result. The amount of AsA was significantly affected by coating and storage time. Although the amount of AsA in all treatments decreased during storage from days 14 to 28, GT significantly reduced the loss of AsA in pepper samples. After 28 days of storage, the AsA of BPFs coated with 1% GT was 82.28% (95.36 mg 100 g^−1^ FW) compared to the control samples, which preserved 59.90% (68.99 mg 100 g^−1^ FW) of the initial amount of AsA.

### 3.12 Antioxidant enzyme activities

#### 3.12.1 SOD

**FIGURE 6:**
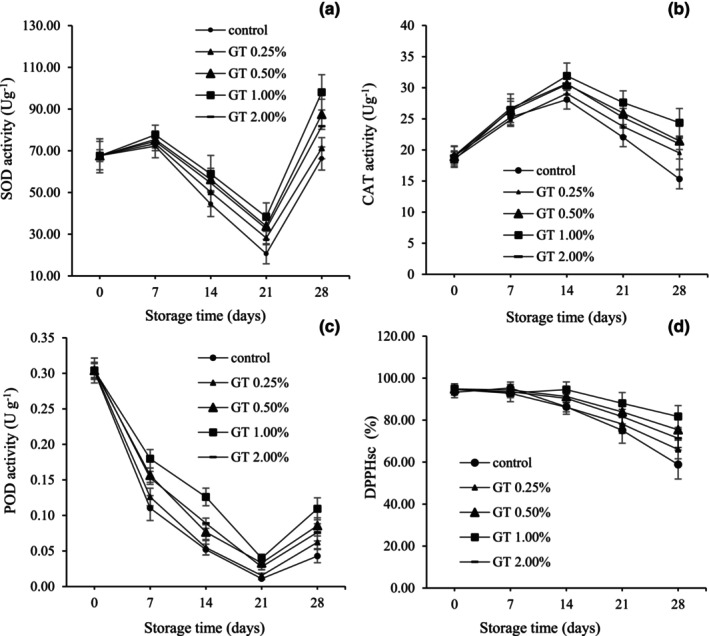
Enzyme activities of SOD (a), CAT (b), POD (c) and total antioxidant activity (d) in pepper fruits coated with GT (0.00, 0.25, 0.50, 1.00, and 2.00%). The fruits were stored at 7°C, 95% RH, for 28 days. LSD_0.05_ indicates the least significant difference (*p* < .05). Vertical bars represent the standard deviation (SD) (*N* = 3).

As seen in Figure 6a, the SOD activity initially increased until day 7, decreased until day 21, and increased again until the end of storage. This trend was similar in all treatments, but in general, coated fruits showed higher SOD activity throughout the period, which was in line with the increase of coating gel up to 1%. At the end of storage, the uppermost level of the SOD engagement in BPFs coated with 1% GT was 122.53 U g^−1^, and the lowermost was observed in uncoated fruits, which was 82.86 U g^−1^.

#### 3.12.2 CAT

The CAT activity in uncoated and coated fruits decreased during 28 days of storage (Figure 6b). Until the 21st day, the highest level of CAT activity was related to the control, and after that, a sharp decrease was observed so that on the 28th day, the lowest amount of CAT was found in the uncoated (control) sample. The CAT activity in peppers coated with GT (1%) on the 28th day was 56.5% higher than uncoated fruits.

#### 3.12.3 POD

As presented in Figure 6c, the POD activity in uncoated and coated fruits decreased until the 21st day of storage and then amplified until the 28th day (Figure 6c). In the entire storage period, the POD activity in coated fruits was superior to that of uncoated BPFs, and this trend was in line with the increase in coating quality.

### 3.13 Total antioxidant content (TAC)

The TAC based on the DPPH radical inhibitor activity was the highest on the 7th day of storage in the control and then decreased with increasing storage life (Figure 6d). TAC had no significant difference in GT‐coated fruits (1%) from the beginning of the experiment to day 14 and began to decline after day 14, but had the lowest rate of decline compared to other treatments. In general, the coated fruits had a higher TAC than the uncoated BPFs at the end of storage.

### 3.14 Lipid peroxidation (MDA content)

**FIGURE 7:**
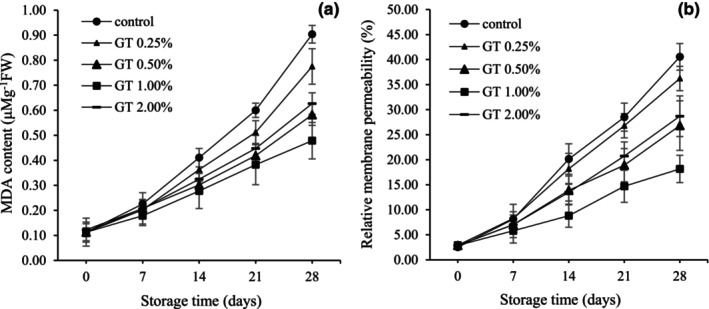
Lipid peroxidation (MDA) (a) and relative membrane permeability (b) in pepper fruits coated with GT (0.00, 0.25, 0.50, 1.00, and 2.00%). The fruits were stored at 7°C, 95% RH, for 28 days. LSD_0.05_ indicates the least significant difference (*p* < .05). Vertical bars represent the standard deviation (SD) (*N* = 3).

Figure 7a shows an increase in the MDA content in all treatments during 28 days of storage. However, using GT‐coated BPFs significantly showed a delayed process for increasing MDA. At the end of storage, the MDA of the samples coated with 1% GT was 47.67% lower than that of the control.

### 3.15 Relative membrane permeability (RMP)

As shown in Figure 7b, the change in leakage of electrolytes from the membrane increased with increasing storage time in all treatments. The RMP in uncoated fruits was significantly higher than in coated fruits. The lowest level of RMP in 1% GT‐coated fruits was 18.15%, which was 55.24% less than the control.

## 4 DISCUSSION

In this study, GT‐coated samples showed less PWL than the uncoated samples. Furthermore, increasing the coating concentration up to 1% affected more maintaining the fruit weights, and the higher concentration (2%) again increased the weight loss. Similar inhibitory effects of GT on fruit weight loss in tomatoes (Jahanshahi et al., 2023), apricots (Ali et al., 2015; Ziaolhagh & Kanani, 2021), and mushrooms (Mohebbi et al., 2012) when used with different concentrations or in combinations with other coatings have been reported. The PWL of fruits and vegetables is due to the water reduction caused by active metabolic procedures, such as respiration and transpiration (Dhall, 2013; Ullah et al., 2017). Lower PWL in coated fruits is due to the effects of the coating as a semi‐permeable physical barrier against moisture escape and oxygen and carbon dioxide exchanges, leading to reduced respiration and water loss (Ali et al., 2015; Dhall, 2013). Reducing the carbon dioxide output from the product and the entry of oxygen into the product reduces ethylene production. As a result, it delays senescence, increases shelf life, and preserves product quality. The type and amount of coating affect the amount of change in the internal atmosphere of the product (O_2_, CO_2_) and the amount of weight loss (Dhall, 2013).

Pepper fruits coated with 1% GT showed more firmness than uncoated ones during storage. It has been reported that the softening enzymes, including pectin esterase and polygalacturonase, change the cell wall and soften the fruit tissue (Manganaris et al., 2005). It has been stated that the softening of bell peppers is related to the destruction of the middle lamella of cortical parenchyma cells and the increase in pectin dissolution, minor changes in pectin molecular weight, and hemicellulose content reduction (Nasiri et al., 2019). As illustrated by Abad Ullah et al. (2017), ECs likely inhibit cell wall softening enzymes by decelerating metabolic actions, resulting in firmer tissue. In addition, the ECs may increase the resistance of BPFs against compositional alterations in the cell wall, therefore retaining moisture and delaying fruit softening. Our findings are consistent with Ziaolhagh and Kanani (2021) on apricots and Mohebbi et al. (2012) on mushrooms, who reported that GT as a palatable coating significantly preserves the firmness of the fruit texture.

Pepper fruits coated with 1% GT had the highest marketability score until the end of the storage period, while the uncoated samples showed a 60% decrease in marketability. As reported by other researchers (El‐Gioushy et al., 2022; Liguori et al., 2021; Shehata et al., 2023), ECs positively affect the overall appearance of the fruit during storage. Maintaining the appearance and quality of the fruit can be related to the effect of EC on preventing moisture loss and preserving the color and composition of the fruit (Kumar et al., 2021). The ECs are a semi‐permeable barrier to prevent moisture loss and gas exchange (CO_2_ and O_2_) on the fruit surface, which reduces water loss, respiration rate, metabolism, and deterioration due to increased enzymatic activity and microbial rot (Velickova et al., 2013). In addition, coatings can reduce the physiological deterioration and spoilage of fruits by increasing the activity of antioxidant enzymes and their ability to inhibit free radicals during storage (Wang & Gao, 2013).

An increase in the TSS content of all BPFs was observed throughout storage, but this increase was significantly lower in 1% GT‐coated fruits than in the control. The increase in TSS is mentioned as an indicator of the ripening and senescence of fruits. The TSS of pepper fruits increases with the enhancement of fruit ripening due to the degradation or biosynthesis of more polysaccharides and the accumulation of sugars (Antoniali et al., 2007). In this research, it was observed that the TSS of coated fruits was lower. The lower upsurge in TSS of coated BPFs may be due to reduced water loss and volatile compounds (Ochoa‐Reyes et al., 2013) and delayed ripening (Ullah et al., 2017). These findings were consistent with those of other researchers on the effects of GT on TSS levels (Abebe et al., 2017; Mohebbi et al., 2012; Ziaolhagh & Kanani, 2021).

The results revealed that as the storage time increased, the TA in both coated and uncoated BPFs decreased significantly. Besides, the application of ECs after harvesting maintained a higher TA than the control. The fast decrease in TA in uncoated fruits is probably owed to higher respiration and oxidation of organic acids, while higher TA in coated fruits could be the result of lower respiration, which finally prevents oxidation of organic acids (Ullah et al., 2017; Ziaolhagh & Kanani, 2021). Previous findings showed that the GT coating leads to higher TA in apricots (Ziaolhagh & Kanani, 2021) and tomatoes (Abebe et al., 2017; Jahanshahi et al., 2023), which is in line with the findings of this research.

The fruit taste is affected by TSS and TA and their ratios. The MI determines the taste and nutritional properties of the fruit. In the present study, TSS exhibited an upward tendency and TA showed a downward tendency with the progress of the storage period, and these trends were less than in the bell pepper fruits coated with GT1%. As a result, the MI also changed less in these treated fruits. These lower changes in TSS and TA could be due to delayed ripening and senescence for lower respiration or reduced moisture loss, and for the protective effects of these coatings (Ullah et al., 2017). The results were consistent with those of other studies on tomatoes (Abebe et al., 2017) and strawberries (Aitboulahsen et al., 2018).

The results showed that bell pepper fruits coated with GT 1% had fewer pH changes during the storage time. The variations in pH are mostly based on changes in organic acids. The concentration of these acids decreases during ripening, and this decrease can be associated with an increase in the rate of respiration, which is likely to use titratable acids as a respiratory substrate (Antoniali et al., 2007; Samira et al., 2013). It has been suggested that higher fruit acidity is an advantage that causes less spoilage (Mohammed et al., 1999). The current result is consistent with previous reports that showed that immediately after harvest, the total acidity of pepper fruits increased and then decreased during storage (Morales‐Castro, 2002; Nasiri et al., 2019; Samira et al., 2013). Furthermore, the significant effect of GT on pH in this study was consistent with the results of other researchers on tomatoes (Jahanshahi et al., 2023) and apricots (Ziaolhagh & Kanani, 2021).

The TSC of fruits was meaningfully affected by GT coating and the storage period. There was an increase in TSC during a prolonged storage period for all treatments. The findings of this study are in contrast to those of Shehata et al. (2023), which stated a decrease in soluble sugars during storage in peppers. The lower TSC in fruits during storage by GT as a palatable coating may be related to the reduction in respiration rate and enzymatic actions of these substances, which leads to a decrease in the breakdown of polysaccharides into simple and soluble carbohydrates during storage (Antoniali et al., 2007).

TCC in BPFs increased with increasing storage period, especially in the control. The color change in peppers could be due to the transformation of chloroplast to chromoplast and changes in the pigment content of BPFs with the progress of ripening. Furthermore, the reduction in red color during storage is due to the progress in ripening and the formation of carotenoids (Ullah et al., 2017; Shehata et al., 2023; Saleh, 2020). The lower increase of TCC in pepper fruits coated with 1% GT at the end of storage describes the ability of ECs to retard the breakdown and synthesis of these pigments (Ullah et al., 2017). These findings obviously express the efficiency of ECs in enhancing the visual quality of BPFs. Our results are consistent with those of other researchers (Ali et al., 2015; Gholamipour Fard et al., 2009; Shehata et al., 2023; Ullah et al., 2017), who reported that discoloration was delayed in fruits coated with ECs due to reduced respiration.

The TPC in bell pepper fruits showed a significant decrease at the end of storage. At the end of storage, the coated BPFs with 1% GT showed the lowest level of changes in the content of total phenolic compounds. The reduction in TPC in the control compared to coated fruits during storage is most likely owing to the higher enzymatic activity of PPO in pepper fruits (Ajmal et al., 2022; Kumar et al., 2021). The findings of other researchers about BPFs (Kumar et al., 2021; Taheri et al., 2020) supported the present results. They reported that ECs retained TPC in BPFs during storage, possibly by delaying ethylene production, lessening lipid oxidation, and controlling enzyme reactions (Ajmal et al., 2022; Kumar et al., 2021).

The amount of AsA gradually decreases during storage. This decrease could be due to increased respiration and oxidation of acids to sugar (Ullah et al., 2017). The present study showed that the BPFs coated with 1% GT were more effective in reducing the loss of AsA as compared to other treatments. Our results are consistent with those of other researchers who showed that ECs were effective in reducing the loss of AsA in BPFs (Adetunji et al., 2019; Hedayati & Niakousari, 2015; Ullah et al., 2017). The use of ECs may reduce the release of oxygen, thus reducing the speed of fruit ripening; thus, it preserves AsA content and delays fruit senescence (Adetunji et al., 2019; Xing et al., 2011). Some researchers showed that ECs can increase the level of CO_2_ and decrease the level of O_2_ around the fruit, thereby helping to prevent the oxidation of AsA (Amal et al., 2010).

As can be seen, BPFs treated with 1% GT had higher SOD, POD, and CAT activities during storage (Figure 5). When senescence appears, it seems to be related to the defense system, comprising antioxidant enzymes such as CAT, SOD, and POD and non‐enzymatic antioxidants (Xing et al., 2011; Xu et al., 2009). These findings were consistent with those of previous research regarding the effect of chitosan coating on the activity of antioxidant enzymes in BPFs (Ajmal et al., 2022; Xing et al., 2011). SOD, CAT, and POD are significant enzymes for detoxicating free oxygen radicals in plant tissues (Xu et al., 2009). The decline in enzyme activity may be related to the reduction in the ability to preclude destruction, and ECs induce the activity of antioxidant enzymes and possibly can improve the protection system of fruits and vegetables (Meng et al., 2008).

Antioxidant substances in food neutralize many oxidation processes caused by free radicals, control tissue damage, and reduce the possibility of devastation of functional and nutritional properties (Kumar et al., 2021). During the storage time, a decreasing trend in TAC was observed. The lowest reduction in TAC was recorded in BPFs coated with 1% GT. The results showed that TG can help prevent the worsening of BPFs during storage by maintaining TAC. The results of this study were in agreement with previous research about the effect of edible coating on the TAC of BPFs (Kumar et al., 2021).

The MDA content and the relative amount of electrolyte efflux are applied as direct and indirect indexes of membrane damage. MDA is frequently utilized as an indicator of cellular oxidative destruction because it is a product of lipid peroxidation (Xu et al., 2009). Electrolyte efflux is usually regarded as an indirect measure of membrane damage in cells caused by adversarial conditions and tissue senescence (Jiang et al., 2001). A continuous increase in MDA and electrolyte leakage was observed for all treatments during pepper fruit storage. However, a 1% GT coating for bell peppers significantly reduced the increase in MDA and electrolyte efflux. As observed in coated fruits, TG reduces the activities of SOD, CAT, POD, and other antioxidant compounds such as ascorbic acid, total phenol, and antioxidant capacity compared to uncoated fruits during storage. As a result, the oxidative stress reduced the amount of MDA and the leakage of electrolytes. These findings are in agreement with the results of other researchers about the effect of ECs on reducing the amount of MDA and electrolyte leakage in bell peppers (Ullah et al., 2017; Xing et al., 2011).

## 5 CONCLUSION

The GT coating is a promising alternative treatment to increase the shelf life of bell peppers. After storage at 8°C for 28 days, the coated samples treated with the GT had a higher percentage of marketable peppers with lower physiological weight loss and more firmness. TSC, TCC (color changes), TPC, and AsA were better preserved in fruits coated with 1% GT. Enzymatic antioxidant activities and TAC were induced in GT‐coated fruits. The values of RPM and MDA in the sample treated with GT were much lower than those without coating during the storage period. Regarding the higher shelf life of coated BPFs, it may be said that GT can be considered as an innovative coating for commercial applications during the storage and marketing of BPFs. This is due to the affordability of GT as compared to chitosan and other ECs, and its effectiveness in low concentrations.

## AUTHOR CONTRIBUTIONS

**Mohammad Reza Zare‐Bavani:** Conceptualization (equal); data curation (equal); formal analysis (equal); funding acquisition (lead); investigation (equal); methodology (equal); project administration (lead); resources (equal); software (equal); supervision (equal); validation (equal); visualization (equal); writing – original draft (lead); writing – review and editing (lead). **Mostafa Rahmati‐Joneidabad:** Conceptualization (equal); data curation (equal); formal analysis (equal); investigation (equal); methodology (equal); resources (equal); software (equal); supervision (equal); validation (equal); visualization (equal); writing – review and editing (equal). **Hossein Jooyandeh:** Conceptualization (equal); data curation (equal); formal analysis (equal); investigation (equal); methodology (equal); resources (equal); software (equal); supervision (equal); validation (equal); visualization (equal); writing – review and editing (equal).

## FUNDING INFORMATION

The work was supported by the Research Affairs of the Agricultural Sciences and Natural Resources University of Khuzestan (Grant No. 928/411/1).

## CONFLICT OF INTEREST STATEMENT

None declared.

## Data Availability

The data that support the findings of this study are available on request from the corresponding author.
